# GLP-1 receptor agonists in metabolic dysfunction-associated steatohepatitis: protocol for a systematic review and meta-analysis of randomized controlled trials

**DOI:** 10.3389/fmed.2026.1800909

**Published:** 2026-09-28

**Authors:** Shi-ping Sun, Yan Fang, Li Liu, Yi Li, Han Min

**Affiliations:** 1 Department of Gastroenterology, The Affiliated Suzhou Hospital of Nanjing Medical University, Suzhou Municipal Hospital, Gusu School, Nanjing Medical University, Suzhou, Jiangsu, China; 2 Department of Gastroenterology, The Affiliated Suzhou Hospital of Nanjing Medical University (Suzhou Municipal Hospital, Suzhou Women and Children's Health Hospital), Suzhou, Jiangsu, China

**Keywords:** metabolic dysfunction-associated steatohepatitis, glucagon-like peptide-1 receptor agonists, systematic review, meta-analysis, randomized controlled trials, trial sequential analysis, liver fibrosis

## Abstract

**Background:**

Metabolic dysfunction-associated steatohepatitis (MASH) is a major global chronic liver disease without approved pharmacotherapy. Glucagon-like peptide-1 receptor agonists (GLP-1 RAs) show therapeutic promise due to their multi-faceted metabolic benefits, but their efficacy on histological endpoints in MASH requires definitive synthesis.

**Methods and analysis:**

We will conduct a systematic review and meta-analysis of randomized controlled trials (RCTs) evaluating GLP-1 RAs versus placebo/standard care in adults with MASH. Literature searches will be performed in PubMed, Embase, Cochrane Library, and Web of Science. Two reviewers will independently screen studies, extract data, and assess risk of bias using Cochrane's ROB 2 tool. The primary outcome is histological improvement (MASH resolution/fibrosis reduction). Secondary outcomes include changes in liver enzymes, metabolic parameters, and adverse events. Data will be pooled using random-effects models. Trial Sequential Analysis (TSA) will be performed to control random errors and evaluate evidence robustness. Subgroup and sensitivity analyses are planned.

**Ethics and dissemination:**

Ethical approval is not required because this study is a secondary analysis of existing data. We will disseminate the findings through peer- reviewed publications.

**Systematic Review Registration:**

PROSPERO CRD420261287573.

## Introduction

Metabolic dysfunction-associated steatohepatitis (MASH) represents an advanced form of metabolic-associated fatty liver disease (MAFLD) and has emerged as a leading cause of chronic liver disease worldwide (1–3). Its prevalence has risen in tandem with the global epidemics of obesity and type 2 diabetes, constituting a significant public health burden. Patients with MASH not only face long-term risks of liver fibrosis, cirrhosis, and hepatocellular carcinoma, but also exhibit markedly elevated all-cause mortality, with cardiovascular disease being the primary cause of death (4–8). Consequently, managing MASH has become a complex metabolic challenge involving multiple systems.

Currently, first-line treatment for MASH relies on lifestyle interventions, but these face limited long-term adherence and inconsistent efficacy (9–12). Although resmetirom has recently received regulatory approval for MASH with fibrosis and semaglutide has shown promising biopsy-based results, globally approved pharmacotherapies remain scarce, and the role of GLP-1RAs in particular has not been conclusively established. This substantial unmet clinical need has spurred researchers to explore the therapeutic potential of drugs already used for other metabolic disorders. This substantial unmet clinical need has spurred researchers to explore the therapeutic potential of drugs already used for other metabolic disorders. Among these, glucagon-like peptide-1 receptor agonists (GLP-1RAs) have emerged as a focal point due to their broad multi-efficacy (13–15).

Beyond effective blood glucose control and weight reduction, GLP-1 receptor agonists have been found to potentially exert direct benefits on the liver (16–18). Their hepatoprotective mechanisms operate through dual pathways: a direct pathway involves inhibiting lipid synthesis and promoting oxidation via receptors on hepatocytes, while potentially suppressing hepatic stellate cell activation; an indirect pathway reduces inflammatory drivers by improving systemic insulin resistance, reducing body weight, and modulating the gut-liver axis. These mechanisms provide a robust theoretical basis for applying GLP-1RAs to MASH. While semaglutide trials have incorporated liver biopsies, many other studies have relied on non-invasive markers, leaving the direct impact of GLP-1RAs on histological fibrosis still uncertain and warranting a dedicated synthesis of biopsy-based evidence (19–21).

However, clinical evidence regarding the impact of GLP-1 receptor agonists on MASH histological endpoints remains inconclusive. Existing randomized controlled trials exhibit limited sample sizes, particularly for hard endpoints such as improvement in liver fibrosis. Published systematic reviews demonstrate heterogeneity in patient populations, control group designs, and outcome definitions, thereby increasing the uncertainty of conclusions. Furthermore, traditional meta-analyses may fail to adequately control for random errors arising from multiple analyses and struggle to determine whether the existing evidence is sufficient (22, 23). Therefore, there is an urgent need for a more methodologically rigorous evidence synthesis study. This study aims to integrate high-quality randomized trial evidence through systematic review and meta-analysis, incorporating trial sequential analysis to assess the reliability of conclusions. The objective is to clarify the precise efficacy and safety of GLP-1RAs in patients with MASH, providing high-level evidence-based guidance for clinical decision-making and future research.

Given that several recent meta-analyses have reported mixed results, our study will differentiate itself by strict inclusion of biopsy-confirmed MASH, pre-specified histological primary endpoints, and the use of Trial Sequential Analysis to determine whether the current evidence is conclusive. Furthermore, we will include the most recently published RCTs and provide a GRADE-based certainty assessment, thereby offering a more robust and up-to-date synthesis.

## Methods

### Protocol and registration

This study will be conducted as a systematic review and meta-analysis, incorporating Trial Sequential Analysis (TSA), in accordance with the Preferred Reporting Items for Systematic Reviews and Meta-Analysis Protocols (PRISMA-P) statement. The review protocol was registered prospectively on the PROSPERO international database (Registration ID: CRD420261287573) (24).

### Eligibility criteria (PICOS)

#### Participants

Adult patients (≥18 years) diagnosed with MASH confirmed by liver biopsy (histological assessment). Studies using only imaging-based diagnosis (e.g., MRI-PDFF, transient elastography) without biopsy will be excluded from the primary analysis; they may be explored in a separate sensitivity analysis if data allow.

#### Intervention

Treatment with any glucagon-like peptide-1 receptor agonist (GLP-1 RA) (e.g., liraglutide, semaglutide, exenatide, dulaglutide) at any administered dose, frequency, or duration.

#### Comparison

Placebo or standard care (including lifestyle interventions, other pharmacotherapies, or no treatment).

### Outcomes

#### Primary outcome

Histological improvement, defined as either resolution of steatohepatitis without worsening of fibrosis or improvement in fibrosis stage (≥1 stage reduction), as assessed by paired liver biopsies.

#### Secondary outcomes

Changes in liver enzyme levels (ALT, AST), metabolic parameters (body weight, HbA1c, lipid profile), incidence of adverse events, quality of life, and imaging-based liver fat reduction.

### Study design

Only published peer-reviewed randomized controlled trials (RCTs) will be considered for inclusion.

### Exclusion criteria

Non-randomized studies, observational cohorts, case reports, reviews, editorials, and conference abstracts; studies where the population included patients with other chronic liver diseases as the primary etiology (e.g., viral hepatitis, autoimmune hepatitis); studies where GLP-1 RAs were used in combination with other investigational drugs for MASH without a GLP-1 RA monotherapy arm for comparison will be excluded from this systematic review and meta-analysis.

### Literature sources and retrieval strategy

A systematic literature search will be designed and executed by an experienced medical librarian. The following electronic databases will be: PubMed, Embase, Cochrane Library, and Web of Science. The search strategy employed a combination of controlled vocabulary (MeSH in PubMed, Emtree in Embase) and free-text terms related to the key concepts: “Glucagon-Like Peptide-1 Receptor Agonists,” “Non-alcoholic Steatohepatitis,” “Metabolic Dysfunction-Associated Steatohepatitis,” and “Randomized Controlled Trial.” The search will be restricted to human studies but had no language restrictions. An example is: (“Glucagon-Like Peptide-1 Receptor Agonists"MeSH OR “GLP-1RA"tiab OR “semaglutide"tiab OR “liraglutide"tiab OR “dulaglutide"tiab OR “exenatide"tiab) AND (“Metabolic Dysfunction-Associated Steatohepatitis"MeSH OR “MASH"tiab OR “non-alcoholic steatohepatitis"tiab OR “NASH"tiab) AND (“Randomized Controlled Trial"pt OR “randomised"tiab OR “randomized"tiab).” We also mention that the same filter will be adapted for other databases

### Literature screening and data extraction

All identified records will be imported into EndNote software for deduplication and screening. The study selection process will be performed independently by two reviewers in two stages: 1) screening of titles and abstracts against the eligibility criteria, and 2) assessment of full-text articles for final inclusion. Any discrepancies at either stage will be resolved through discussion or by adjudication from a third senior reviewer. A standardized, piloted data extraction form will be used to collect data from included studies. Extracted information includes: (a) Study characteristics (first author, publication year, country, funding source, trial design, duration); (b) Participant characteristics (sample size, age, sex, BMI, diabetes status, baseline liver histology/biochemistry); (c) Intervention details (type of GLP-1 RA, dose, frequency, administration route); (d) Comparator details; (e) All pre-specified primary and secondary outcomes with their definitions and measurement timepoints; (f) Results for each outcome. Data extraction will be performed independently by two reviewers, and consistency will be checked.

### Risk of bias and certainty of evidence assessment

The methodological quality of each included RCT will be assessed independently by two reviewers using the revised Cochrane Risk of Bias tool for randomized trials (RoB 2.0). The tool evaluates bias across five domains: bias arising from the randomization process, bias due to deviations from intended interventions, bias due to missing outcome data, bias in measurement of the outcome, and bias in selection of the reported result. Each study will be judged to have “low risk,” “some concerns,” or “high risk” of bias for each domain and an overall judgment. Disagreements will be resolved by consensus. The overall certainty (quality) of evidence for each primary outcome will be evaluated using the Grading of Recommendations Assessment, Development and Evaluation (GRADE) approach, considering risk of bias, inconsistency, indirectness, imprecision, and publication bias.

### Data synthesis and statistical analysis

#### Meta-analysis

Statistical analysis will be performed using Review Manager (RevMan, version 5.4). For dichotomous outcomes, risk ratios (RRs) with 95% confidence intervals (CIs) will be calculated. For continuous outcomes, mean differences (MDs) or standardized mean differences (SMDs) with 95% CIs will be calculated, depending on whether outcomes are measured on the same scale. Statistical heterogeneity will be assessed using the I^2^ statistic and Cochran's *Q* test. An I^2^ value > 50% or a significant Q test (*p* < 0.10) indicates substantial heterogeneity. A random-effects model (DerSimonian and Laird method) will be used as the primary analysis model to account for anticipated clinical and methodological heterogeneity. A fixed-effect model will be also applied as a sensitivity analysis. For missing standard deviations, we will attempt to calculate them from confidence intervals, p-values, or standard errors; if only medians and ranges are reported, we will apply the method of Wan et al. to approximate means and SDs. When outcomes are measured on the same scale across studies, we will use mean difference (MD); otherwise, standardized mean difference (SMD) will be used. If at least 10 studies are included, we will conduct random-effects meta-regression using the pre-specified subgroup characteristics to examine sources of heterogeneity.

#### Trial sequential analysis (TSA)

TSA will be performed for the primary histological outcome using TSA software (version 0.9.5.10 Beta). The required information size (RIS) will be calculated based on a two-sided alpha of 5%, beta of 20% (80% power), and the pooled control event proportion observed in the included trials. We will assume a relative risk reduction of 20-25% for MASH resolution or fibrosis improvement, as informed by prior trials. The Lan-DeMets alpha-spending function with O'Brien-Fleming boundaries will be applied. If substantial heterogeneity is detected (I^2^ > 50%), we will apply a diversity adjustment when computing the RIS. The monitoring boundaries will determine whether the cumulative evidence is sufficient to accept or reject the effect.

#### Subgroup and sensitivity analyses

Pre-specified subgroup analyses will be conducted to explore potential sources of heterogeneity: 1) Type of GLP-1 RA (semaglutide, liraglutide, other) and formulation (oral vs. injectable); 2) Treatment duration (≤6 months vs. >6 months); 3) Presence of type 2 diabetes at baseline; 4) Baseline fibrosis stage (F0-F2 vs. F3-F4); 5) Dose (high vs. low as defined by trial). If sufficient data are available, we will also compare biopsy-confirmed versus imaging-based studies in a separate sensitivity analysis.

#### Assessment of publication bias

If 10 or more studies are included in a meta-analysis, publication bias will be assessed visually using funnel plots and formally tested using Egger's regression test.

## Discussion

This study aims to comprehensively evaluate the efficacy and safety of glucagon-like peptide-1 receptor agonists in treating metabolic dysfunction-associated steatohepatitis through a systematic review and trial-sequential analysis. Currently, despite the growing recognition of GLP-1RAs in metabolic disease treatment, definitive conclusions based on high-quality randomized controlled trials regarding their precise impact on core MASH histological endpoints—such as steatohepatitis resolution and fibrosis improvement—remain lacking (20–23). Existing evidence is fragmented and heterogeneous, and traditional reviews struggle to overcome risks of statistical error due to insufficient sample sizes and multiple analyses (22, 23). Therefore, this study aims to rigorously synthesize relevant randomized controlled trials worldwide, focusing not only on core efficacy measures like liver histology, serology, and imaging but also systematically evaluating their impact on metabolic parameters and overall safety profiles. Crucially, we will employ the advanced statistical method of sequential analysis to determine whether the existing cumulative evidence is sufficient and reliable to provide conclusive evidence-based support for the clinical application of GLP-1RAs in MASH. Simultaneously, this approach will precisely identify evidence gaps, guiding the design of future large-scale clinical trials.

Several systematic reviews and meta-analyses have evaluated GLP-1-based therapies in MASH, but they often included mixed populations, varied outcome definitions, and did not apply Trial Sequential Analysis. Our study differs by strictly restricting to RCTs with biopsy-confirmed MASH as the primary analysis, using TSA to control random errors, and incorporating the most recent trials. This approach aims to provide a more robust and definitive estimate of the true effect on histological endpoints.

This study design demonstrates significant methodological rigor and innovative advantages. First, the entire research process will be planned and reported in strict adherence to the PRISMA-P guidelines, with prospective international registration already completed. This ensures transparency, reproducibility, and control over publication bias in the study design. The most critical advantage lies in the planned use of trial sequential analysis. Traditional meta-analyses, when continuously updating accumulated evidence, may increase the risk of Type I errors (false positives) due to repeated significance testing. TSA effectively corrects this random error by pre-calculating the required information size and constructing monitoring boundaries, enabling a more scientific and robust determination of whether evidence is sufficient. This means our study not only answers whether GLP-1RAs are effective but also further assesses the reliability of this conclusion—whether it is conclusive or remains in the “false positive” or “uncertain” zone requiring more evidence. The application of this method will significantly enhance the robustness of this meta-analysis's conclusions and its guidance value for clinical decision-making.

Second, this study employs a predefined analytical framework that is both in-depth and highly clinically relevant to maximize the value of extracted information. We will conduct comprehensive subgroup analyses to explore the potential impact of key factors on efficacy, including different GLP-1 receptor agonist drug types (e.g., long-acting versus short-acting formulations), treatment duration, presence of concomitant type 2 diabetes, and baseline liver fibrosis severity. This analysis will help elucidate potential sources of therapeutic heterogeneity, providing preliminary insights for future precision treatment strategies targeting distinct MASH patient subgroups. Additionally, we will employ the internationally recognized GRADE evidence quality grading system to assign “high, moderate, low, or very low” ratings to the evidence body for each primary outcome. This approach not only systematically assesses risks of bias, inconsistency, indirectness, imprecision, and publication bias but also enables clinicians and policymakers to intuitively grasp the certainty of our final conclusions, thereby facilitating more prudent weighing of treatment benefits against risks.

This study may also have several limitations. First, the strength and generalizability of the analytical conclusions fundamentally depend on the number, quality, and clinical characteristics of the original studies ultimately included. If there are too few high-quality randomized controlled trials meeting the criteria, it may weaken the statistical power of the pooled analysis and limit the generalizability of the conclusions. Second, differences among individual studies in MASH diagnostic criteria (e.g., biopsy vs. imaging), follow-up duration, and control group interventions (e.g., intensity of lifestyle guidance) may introduce clinical and methodological heterogeneity. This variability may not be fully accounted for by statistical models, posing challenges for interpreting pooled results. Furthermore, as a secondary study based on published literature, we lack access to individual patient-level data. This limitation restricts deeper explorations, such as evaluating dose-response relationships, analyzing associations between specific biomarkers and treatment responses, or conducting more detailed patient subgroup analyses. Future research directions should include conducting rigorously designed, adequately powered randomized controlled trials with longer follow-up periods (to assess hard endpoints such as cirrhosis and hepatocellular carcinoma). Additionally, encouraging researchers to share data to support meta-analyses based on individual patient data will help validate and deepen the findings of this study, ultimately advancing the optimization and individualization of MASH treatment strategies.
